# The Formation and Transformation of Medical Apartheid in Palestine: A Historical Examination

**DOI:** 10.1177/27551938251380186

**Published:** 2025-09-25

**Authors:** Osama Tanous, Yara M. Asi, Bram Wispelwey, David Mills, Weeam Hammoudeh, Rania Muhareb

**Affiliations:** 1 FXB Center for Health and Human Rights, 580389 Harvard University, Boston, USA; 2 School of Global Health Management and Informatics, 6243 University of Central Florida, Orlando, USA; 3 Harvard Medical School, Boston, USA; 4 Department of Pediatrics and Emergency Medicine, 12220 University of California San Diego School of Medicine, La Jolla, USA; 5 Institute of Community and Public Health, Birzeit University, Birzeit, Palestine; 6 The Irish Centre for Human Rights in the School of Law, 8799 University of Galway, Galway, Ireland

**Keywords:** Palestine, medical apartheid, racism, settler colonialism, health disparities, statelessness

## Abstract

Apartheid is clearly defined as a crime against humanity under international law, involving inhuman(e) acts committed in the context of systematic oppression and domination by one racial group over any other. The term apartheid has long been used to describe the experience of the Palestinian people. Despite its increased use in recent years, the term “medical apartheid” has not been as formally defined by public health bodies. In this article, we use a settler colonial lens to track the formation and expansion of health care services in Palestine/Israel that has mirrored the current reality of systematic oppression and domination, where Jewish Israelis and Palestinians across fragmented geographies enjoy differential access to the full enjoyment of their right to health. We examine the development of the health care services accessible to Palestinians to explore larger notions of statehood/statelessness, (denial of) sovereignty, citizenship, de-development, dependency, humanitarianism, and aid as they shape the life, health, illness, and death of Palestinians. By exploring the historical events that led to the formation of separate and unequal health care systems, built by and for different populations in Palestine/Israel, we identify the contours of Israel's medical apartheid system.

Apartheid is a crime against humanity under international law. It is defined in Article II of the 1973 International Convention on the Suppression and Punishment of the Crime of Apartheid as involving “inhuman acts committed for the purpose of establishing and maintaining domination by one racial group of persons over any other racial group of persons and systematically oppressing them”.^1^ Article 7(2)(h) of the 1998 Rome Statute of the International Criminal Court similarly defines the crime of apartheid as: “Inhumane acts . . . committed in the context of an institutionalized regime of systematic oppression and domination by one racial group over any other racial group or groups and committed with the intention of maintaining that regime”.^2^

Despite its increasing use in recent years, the term “medical apartheid” has not been as formally and broadly defined.^3^ In this article, we offer a historical framing of the concept of medical apartheid by tracing the development of such a system, using the context of Palestine.^4–6^

In 2024, the International Court of Justice (ICJ) held that Israel is in breach of the prohibition on racial segregation and apartheid under international law,^7^ while in recent years numerous states,^8^ U.N. bodies and experts,^9,10^ and human rights and civil society organizations have recognized Israeli apartheid against the Palestinian people,^4–6,11^ including South Africa in its ongoing case against Israel under the 1948 Convention on the Prevention and Punishment of the Crime of Genocide.^12^

## Defining Apartheid

The system of apartheid as imposed in southern Africa from 1948–1994 was preceded by centuries of settler colonialism, racial segregation, and domination in South Africa and around the world, the contours of which were generally determined by European colonization of land and labor. Although apartheid violated the central tenets of public international law prior to the establishment of the United Nations in 1945, it was not until 1948 that apartheid was declared a violation of the U.N. Charter and, as a result of the efforts of the anti-apartheid movement during the decolonization era, was gradually prohibited and condemned.^13^

In 1960, the U.N. General Assembly affirmed that the “process of liberation is irresistible and irreversible and that, in order to avoid serious crises, an end must be put to colonialism and all practices of segregation and discrimination associated therewith”.^14^ In the following years, states “particularly condemn[ed] racial segregation and apartheid and undertake to prevent, prohibit and eradicate all practices of this nature in territories under their jurisdiction” in Article 3 of the International Convention on the Elimination of All Forms of Racial Discrimination (ICERD), and the General Assembly labeled apartheid a crime against humanity.^15^ By 1973, the international community adopted the Apartheid Convention, which declared apartheid not only unlawful but criminal toward its suppression and punishment.^1^

Apartheid consists of three main elements:^16^ an intent to maintain domination by one racial group over any other, a context of systematic oppression by one racial group over any other, and inhuman(e) acts committed in this context. In recent years, scholars and international human rights organizations, bolstered by meticulous legal analysis and justification, have since applied it to other contexts, including the Myanmar government's system of discriminatory laws against the Rohingya people.^17,18^ Charges of apartheid have also surfaced elsewhere, notably in relation to the caste systems in India and North Korea,^19^ and have also been levied against the Brazilian state and its treatment of its Black population.^20^

However, the state most extensively and credibly charged with enforcing a system of apartheid is Israel against the Palestinian people. Palestinians have long been calling the system of domination that governs their lives, undermining their right to health and well-being, as one of apartheid.^11^ In 1965, before the start of Israel's military occupation of the West Bank and the Gaza Strip, Fayez Sayegh, then the director of the Palestine Research Center in Beirut, critiqued “the Zionist practitioners of apartheid in Palestine”.^21^ While initially dismissed and censored, the apartheid framework has gained more prominence and attention in mainstream circles of late, as a result of detailed reports published by human rights organizations who have joined this discourse and accepted this framing of the reality on the ground.^5,6,22^

While some reports on Israeli apartheid limit the scope of analysis geographically and temporally to the occupied Palestinian territory since 1967, we concur with those who have insisted that a more comprehensive articulation of the root causes is necessary—one that centers the ongoing *Nakba* (catastrophe) of the Palestinian people since 1948,^23^ situates Israeli apartheid within the broader context of settler colonialism, and considers the experience of the Palestinian people as a whole, including their strategic fragmentation by Israel as a tool of apartheid.^9^ The apartheid report released by Amnesty International in 2022 notably concluded that apartheid applies beyond this geographical scope to the Palestinian people “whether they live in Israel, the Occupied Palestinian Territories (OPT), or in other countries as refugees”.^24^

In addition, the coalition report published by the Palestinian human rights organization Al-Haq framed apartheid as a tool of Zionist settler colonialism,^4^ while the settler colonial determinants of health framework likewise notes apartheid as an intermediary technology of settler colonial elimination.^25^ We build on Yazid Barhoush and Joseph Amon's call for better understanding and defining medical apartheid in the Palestinian context with a view to “build[ing] a consensus for its global elimination”.^3^

## Defining Medical Apartheid: Health as a Manifestation of Apartheid

Unlike the concept of racism and its health effects, which has been increasingly researched over the previous decades,^26,27^ the relationship between apartheid and health remains much less explored, despite the obvious overlaps between the two concepts with apartheid understood as an extreme form of racial discrimination. As a result, the term *medical apartheid* has been given different meanings in different settings.

In the United States, the term *medical apartheid* has been most widely used with reference to unethical medical treatment and experimentation on African Americans^28^ or the adverse effect of segregation and racism on access to health care, insurance coverage, and health outcomes.^29^ In South Africa, where the term apartheid itself originated (meaning “apartness” in Afrikaans), it often refers to the adverse health effects of the apartheid system enacted on people of color in South Africa and the segregation of the health systems that resulted from the broader separation of the races.^30^ The effects of apartheid on the health system inherited by the African National Congress in 1994 have been documented as they relate to the repercussions for public health in South Africa and its ongoing deficiencies, from a “combination of deliberate official policy, discriminatory legislation and at times blatant neglect”.^31^

In 1981, the International Conference on Apartheid and Health was held in People's Republic of the Congo at Brazzaville, with a report released from the conference in 1983. The report, which focused on apartheid in South Africa, described “apartheid as a negation of the right to health,” while also extending “solidarity to all the people fighting against colonialism, racism, Zionism, fascism, neocolonialism and imperialism for national liberation and peace, which are themselves the prerequisites for social progress and health development.” The report cited the many causes of health disparities, notably “lack of political control, inadequate land, poor housing, inappropriate or absent education and unrealistic earning power,” as well as the outcomes, including poor occupational health measures, high infant and maternal mortality rates, low rates of medical practitioners, higher rates of communicable disease, multiple mental health issues, and malnutrition. The conference argued that “the health care system created under apartheid is a faithful image and a particular case of the racial, social, economic inequalities that dominate that society”.^32^ Critically, the Brazzaville Declaration adopted at the conference emphasized the dismantlement of the apartheid regime and the realization of the right to self-determination as “the single essential prerequisite for the establishment of a health care system in South Africa, which would meet the needs of all people and embody the principles of health for all”.^32^

Using evidence from the Palestinian context, in 2023 *medical apartheid* was defined by Barhoush and Amon as “a discriminatory system of policies and practices seeking to reinforce racial or ethnic segregation and resulting in direct harm and stark inequality in health care accessibility, availability, acceptability, and quality.” Specifically, the authors described the underinvestment in Palestinian health (in large part due to Israeli control of the Palestinian economy and borders), the Israeli attacks on health care facilities and workers, and the rates of denials and delays of the Israeli medical permits Palestinians must apply to in order to access care unavailable in Palestinian territories (due to Israeli restrictions) as evidence of a system of medical apartheid.^3^ While Barhoush and Amon limited their analysis to medical apartheid in the occupied Palestinian territory since 1967, the present article expands this discussion by tracing the historical trajectory of Israel's medical apartheid as it manifests in the health care systems accessible to Palestinians and Israelis in the context of settler colonialism.

This article explores, frames, and describes medical apartheid by drawing from the historical trajectory of the development of the state of Israel and the non-sovereign, now-occupied Palestinian territories. In so doing, we hope to both describe the specific practices of the Israeli state that detrimentally impact the right to health of the Palestinians who live under its control as well as contribute to the growing body of literature on medical apartheid, which, like apartheid itself, is a concept that should have universal applicability rather than historical or geographical limitations.^33^

Incorporating insights from settler colonial and Indigenous studies,^34^ we take the opportunity to theorize from a context representing an “unconcealed structure of domination”^35^ to apply the legal framework of apartheid to the health care system(s) accessible to Palestinians and Israelis, in what we term medical apartheid. We follow Erakat and colleagues^33^ in accentuating the importance of the right to self-determination in any definitional debate. Indeed, Palestinian human rights organization BADIL has stressed that Israeli apartheid is “not an end in and of itself but is rather a mechanism designed to eliminate the Palestinian people by undermining their collectivity”.^36^

**Figure 1.**
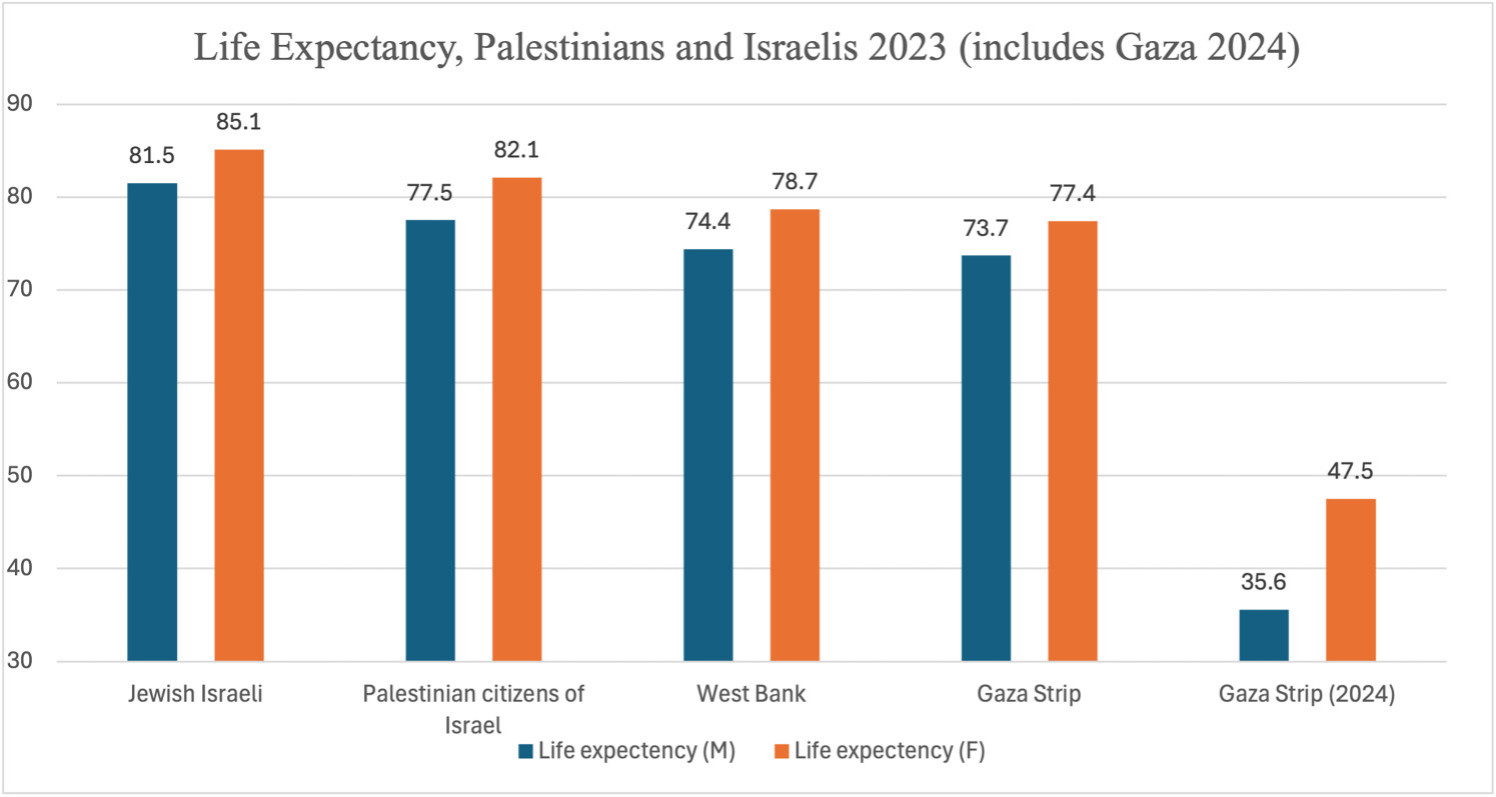
2023 Life Expectancy of Palestinian and Israeli Men and Women, Including the Gaza Strip in 2024.

Rather than defining medical apartheid solely as the disparate racialized health outcomes of an apartheid system, most visible in the gaps in life expectancy of the different groups living between the river and the sea (see Figure 1), we argue for a definition in which health systems (including those of both the oppressor and the oppressed) themselves constitute and reinscribe racial domination, systematic oppression, and inhuman(e) acts. We argue for the need to track the formation and transformation of health care systems as the mirror dynamics of colonial and racial supremacy.

**Figure 2.**
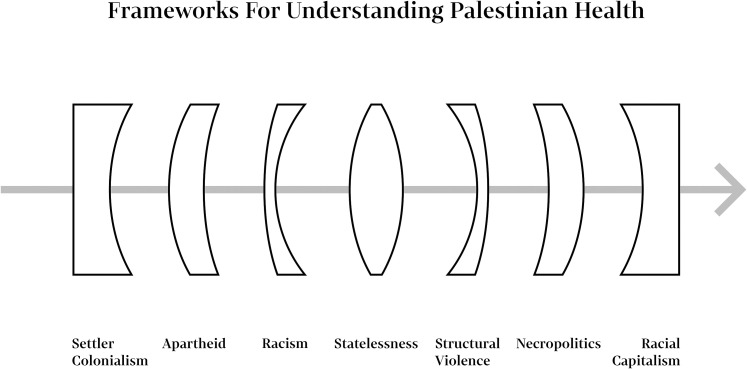
Frameworks for Understanding Palestinian Health.

In this article, we analyze health care as an infrastructure. The health care system, with its vast network of clinics, hospitals, laboratories, and administration, is a life-sustaining infrastructure that makes life imaginable and possible in a certain place. In a settler colonial context, infrastructures like water,^37^ sewage,^38^ waste,^39^ roads,^40^ electricity,^41^ and telecommunications^42^ have been studied as a tool of both life provision and life destruction.^43^ The expansion of health care to certain places makes life possible, and in fact, encouraged for settlers, while the destruction or de-development of health care for native people can make places unlivable and be part of campaigns of ethnic cleansing and genocide.^44^ As we explore and track the different health care systems accessible to Palestinians across time and space, we encourage the reader to think of the life-enabling and life-terminating power of infrastructures and their cumulative impact across successive regimes of control.^42^ Doing so allows us to explore yet another contour of settler colonial violence as it shapes the material and social life of the native. Furthermore, we do not argue that the apartheid framework is the only, or most accurate, tool of analysis for understanding Palestinian health. We think that to fully engage with Palestinian health, and the Question of Palestine more broadly, multiple frameworks, or lenses, can and should be used interchangeably to provide a clearer understanding of reality (see Figure 2).

## The History of Palestinian Health Care: Separate and Unequal

To properly understand how medical apartheid manifests and is experienced by Palestinians today, we must first reflect on the making of Israeli sovereignty and statehood, and the unmaking of Palestine sovereignty and statehood, and its relation to the health care systems accessible to Palestinians today.

For hundreds of years, much of the Levant was ruled by the Ottoman Empire.^45^ For much of this time, social services were extremely poor, as there was very little financial allotment to public health. Infectious disease spread was constant, including regular outbreaks of malaria, cholera, and smallpox.^46^ The first significant improvements in public health did not come until the late nineteenth century, as physician licensing became more common, and advancements in security, trade, water storage and distribution, and communication helped increase living standards. These reforms were part of a push to strengthen the empire's position as it faced both internal and external challenges. However, the area of Palestine, composed primarily of peasants outside of coastal areas and urban centers like Jerusalem, remained underdeveloped even by the standards of the region.^47^

With the collapse of the Ottoman Empire after World War I, the geopolitics of the region changed irreversibly. The victorious French and British empires divided much of the Middle East region in the 1916 Sykes-Picot Agreement into the nation-states we know today. They created the borders of Lebanon and Syria, to be under French Mandate, and Palestine, Jordan, and Egypt under the British Mandate.^45^ It is in this period that we first see the bifurcation of colonial medicine and settler colonial medicine in Palestine, and can detect the roots of a medical apartheid system.

### Colonial Medicine

In Palestine, as in other contexts of colonialism and mandate, colonial hospitals were established to serve the circulating troops and pilgrims and thus were mainly concentrated in urban centers. Such hospitals in Palestine were found in Nazareth, Jaffa, and Jerusalem. However, their main interest was management and elimination of infectious disease, as is common in colonized environments, because they were an immediate threat to the Europeans entering the region.^46^

Unlike Iraqis, Egyptians, or Syrians who were slated to gain independence once the Mandate was over, Palestinians were not the ones planned to gain independence and statehood in Mandate Palestine. Instead, the 1917 Balfour Declaration, issued by the British government, promised Palestine to the burgeoning Zionist movement in order to establish a national home for the Jewish people. Palestinians, the native majority in their homeland, were regarded in the Balfour Declaration simply as “non-Jewish” communities, rootless natives in the land who were not entitled to development or viewed as a people who will inherit the polity.^48,49^

In 1937, in response to the Palestine Royal Commission, Winston Churchill argued, “I do not agree that the dog in a manger has the final right to the manger even though he may have lain there for a very long time. I do not admit that right. I do not admit, for instance, that a great wrong has been done to the Red Indians of America or the black people of Australia. I do not admit that a wrong has been done to these people by the fact that a stronger race, a higher-grade race, a more worldly wise race to put it that way, has come in and taken their place”.^50^ Churchill's statement exemplifies much of the attitude of the British empire toward Palestine and its functioning as an incubator for the Zionist settler colonial movement.^23^ The Mandate period was thus a time when the British invested the bare minimum in Palestinian health care while supporting the Zionist movement to develop its own network of hospitals, clinics, health care services, institutions, and a medical school. The concepts of segregation, competition, and the stifling of the racialized native population was established early.

While the British viewed the incoming Zionist settlers as “fellow colonists,” Israeli historian Ilan Pappé argued they saw the Palestinians as “yet another colonized people who had to be oppressed”.^51^ These colonial and racist forces, coupled with a lack of resources, prevented the development of Palestinian institutions at a time of an increase in institutions created specifically for Palestine's rapidly increasing Jewish population. In particular, Article 4 of the British Mandate for Palestine expressly elevated the status of Zionist institutions to that of a “public body” that would cooperate with the British Mandate toward the “establishment of the Jewish national home . . . [in] the interests of the Jewish population in Palestine”.^52^ These institutions of the Zionist movement were chartered to benefit exclusively persons of “Jewish race or descendancy”.^53^

Both the British Mandate and the growing Zionist interest and presence in Palestine purposely neglected and excluded the Palestinian population who were living there—for example, Palestinians were largely excluded from leadership positions in the British Department of Health.^48,54^ Yet, the native Palestinian population made sustained efforts to provide for themselves, including relying heavily on their own forms of traditional medicine. Additionally, despite the lack of a local medical school, a growing middle class studied medicine and nursing in Beirut, Istanbul, or elsewhere and returned to work in Palestine.^54^ Almost 400 Palestinian doctors worked in Palestine during the Mandate years, most of whom studied at the American University of Beirut. While not organized under a state health system, they were engaged in regional medical efforts. In 1933, the first Congress of Arab Doctors was held in Haifa, and in 1944 a Palestinian-Arab Medical Association was established.^54^ The younger generation of Palestinian doctors were aware of the biased role the British Mandate was taking in supporting Zionist medical institutions and the underdevelopment of the Palestinian health sector. By 1947, these associations had engaged in multiple development and education initiatives, including fundraising for hospitals and other small steps that resembled a nation-building effort. But these efforts were short-lived, as the 1948 Nakba expelled the majority of the Palestinian people from their homeland, delaying their attainment of self-determination and sovereignty.

### Settler Colonial Medicine

The core of the settler colonial project is the appropriation of land, the violent depopulation of the native populations from that land, and attempts to establish a new demographic majority on the land.^55^ Such a massive project requires fit and healthy people to cultivate the land, to build settlements, to train the military, and, generally, to create facts on the ground. Here, parallel to the value attributed to soldiers in the colonies, every settler is of use to the settler colony. Early Zionist public health experts stated early on that every working day is valuable for the greater project of settlement and should not be wasted by diseases or long travels to see a doctor.^56^ The Indigenous people was not part of the target audience or envisioned nation, for according to Theodor Herzl, considered to be the founder of political Zionism, “We shall try to spirit the penniless population [his wording for Palestinians] across the border by procuring employment for it in the transit countries, while denying it any employment in our own country”.^a,58^ The roots of Israel's medical apartheid system lies in this juncture, wherein a group of people, self-defined in racial terms, establish a settler polity on Indigenous land and create a health care system by and for settlers. The establishment and expansion of this settler health care system is only possible when a native population is denied its right to sovereignty, statehood, or the development of its own health care system.

The Zionist movement, confident of its inheritance of the land of Palestine after the end of the British Mandate, began to establish long-term health care infrastructure. In 1911, the Agricultural Laborer Union established the General Sick Funds (currently, Clalit Healthcare Services;^59^). Other health care clinics and insurance bodies (sick funds or Kupat Holim) were established between 1911 and 1941. They followed the German model of sick funds and symbolized a pioneer cooperative effort of mutual aid. The four different sick funds were affiliated with various Zionist labor and agricultural unions. Jewish workers gave part of their monthly salary to the sick fund in return for medical insurance and services. Hospitals were also established by settlers and for settlers with the help of entrepreneurs and the sick funds. The Hadassah Medical Organization, founded in 1921 by the American Zionist women's organization Hadassah, operated clinics, hospitals, midwifery programs, and mother and child clinics in charge of vaccination and children follow-ups.^56,60^ With time, this young health care system became a magnet for European and U.S. Jewish doctors and nurses who were fleeing antisemitism or wanted to join efforts of establishing a Jewish state in Palestine.^61^

In the eyes of the British Mandate, the Jewish settlers were a group seen as a sovereign people, building the infrastructure of a promised state, with all that entails in terms of medical infrastructure, universities, electricity infrastructure, labor unions, and so on. At the same time, the Palestinians were seen as non-sovereign communities, not a “people” who have a right to self-determination and to establish their own state or independent health care system. This human-made separation dictates and shapes much of what we see today as medical apartheid in Palestine.

### The Nakba and Creation of Israel: Deliberate Catastrophe and Fragmentation

In 1948, the Zionist movement declared Israeli statehood and hundreds of thousands of Palestinians were displaced and dispossessed by Zionist militias, resulting in a population-level catastrophe known as the Nakba. Despite the informal parallel system that was being put in place prior to Israel's creation by the Zionist movement, the Nakba represented a major step in codifying the apartheid system as defined by international law: one of domination, oppression, and inhuman(e) acts committed against the Indigenous people of Palestine.^23^ The institutions of the Zionist movement, previously declared a public body by the British Mandate, were given quasi-governmental status under Israeli law with adoption of the 1952 World Zionist Organization – Jewish Agency (Status) Law. The latter defined the “central task” of the Zionist movement and of the Israeli state as that of “gathering in the exiles”,^62^ referring to Jewish nationals whose status is distinct from citizenship under Israeli law.^63^ This constructed status and the accompanying preferential treatment it confers on Jewish nationals at the expense of all others, in particular native Palestinians, lies at the heart of Israel's apartheid legal system.^64^

The Zionist parastatal institutions that have considerable sway on state policy were explicitly chartered to benefit Jews only, notably in the allocation of land largely confiscated from Palestinian refugees since the start of the Nakba in 1948.^65^ It is worth noting, as the U.N. Committee on Economic, Social and Cultural Rights (CESCR) has, that the “large­scale and systematic confiscation of Palestinian land and property by the State and the transfer of that property to these [Zionist] agencies constitute an institutionalized form of discrimination because these agencies by definition would deny the use of these properties to non ­Jews”.^66^

Settler colonialism is fundamentally different from franchise colonialism. The settler society in settler colonial settings does not aim primarily to exploit the Indigenous peoples, but rather to replace them altogether with a society of settlers operating within the “logic of elimination”.^55^ Settlers in what became the settler colonial states of the United States, Canada, Australia, New Zealand, and Israel traveled with their sovereignty, seeking not to integrate within an existing society like migrants or refugees but to establish a new, distinct polity.^25,67^ The Zionist movement also wanted to establish a Jewish state with a Jewish majority, not a settler minority as established, with ill-fated consequences, in settler contexts like South Africa, Rhodesia or colonial Algeria.^68^ In short, it sought the racial *elimination* of Palestinians.^21^ In order to do so, Zionist militias during the Nakba committed multiple massacres, bombing, raiding, and terrorizing entire towns and villages, leading to the mass expulsion of the Palestinian people from their land.^69,70^

The Nakba is of crucial importance for the current system of apartheid and medical apartheid,^34^ where the Palestinian people and geography were strategically fragmented and subjugated.^71^ The denial of their right of return and the expropriation of Palestinian refugee property were institutionalized in Israeli law through, in particular, the 1950 Absentees’ Property Law.^72^ They served both to establish Israel's apartheid system and to fragment Palestinians across domains of Israeli control and in forced exile, and to continuously maintain it. By depriving the Palestinian people of individual and collective rights, including their internationally-recognized rights to self-determination and return, Israeli policies ensure that Palestinians remain subjugated and undermine their efforts to challenge the regime.^9^

The largest fragmented group of Palestinians are refugees who were, and continue to be, denied their right of return to their homeland after it was declared a Jewish state, where “return” is considered an exclusive right of Jewish nationals under the 1950 Law of Return.^70,73^ Palestinian refugees experience varying realities across host countries. Palestinian refugees in Jordan, Syria, and Lebanon receive health care services from the U.N. Relief and Works Agency for Palestine (UNRWA) and different services from the states in which they live. Palestinian refugees in Lebanon suffer perhaps from the worst access to health care in a context dominated by racism and xenophobia by the Lebanese state.^74^ While further examination of the health care of Palestinian refugees in exile is warranted, it is not discussed in this article due to our focus on Israel's medical apartheid system. For this purpose we emphasize, however, that the continued denial of Palestinian refugees’ right of return to their homeland constitutes an inhumane act of Israel's apartheid regime, within the understanding of the Apartheid Convention, and contributes to unlawfully altering the demography of historic Palestine. Realizing this right as well as Palestinians’ collective right of self-determination are thus essential steps in dismantling Israeli apartheid and realizing the right of Palestinians to the highest attainable standard of health.^75^

The group of Palestinians who either remained in their towns or were internally displaced inside the Green Line were subjected to military rule and became Israeli citizens.^76–78^ Some of the Palestinian refugees, dispossessed from their towns within historic Palestine, fled to the West Bank, including east Jerusalem, and the Gaza Strip.^79^ Palestinians in the West Bank and Gaza Strip, areas whose contours were defined by Israel's settler colonial frontier expansion, had to accommodate this massive influx of refugees dispossessed from the areas inside the nascent state of Israel.^80^ Palestinians in occupied East Jerusalem were given a special unique status as “permanent residents” and not as Israeli citizens. Fragmented Palestinians, part of a collective identity, suddenly had to adapt to completely different realities imposed on them by the Israeli regime.^71^

The ongoing Nakba is the process of ongoing fragmentation, dispossession, and displacement of Palestinians as they endure varying shades of settler colonial violence.^23^ The fragmented communities of Palestinians live under Israel's effective control and have different identification cards according to a hierarchical and racist system of stratification.^23,81^ This strategic fragmentation itself, as affirmed by Richard Falk and Virginia Tilley in their 2017 report for the U.N. Economic and Social Commission for Western Asia (ESCWA), operates as a main tool of Israel's apartheid regime. By confining Palestinians to fragmented geographies within historic Palestine and exile, Israel further “obscure[s] this regime's very existence”.^9^

In this fragmented context, different health care systems give different services to the fragmented Palestinian people. Examining the health care systems and services available to those fractured communities against the backdrop of those provided to Jewish nationals helps us understand Israel's medical apartheid and its building blocks.

## The Israeli Health Care System After 1948

After the Nakba of 1948 and the Israeli declaration of statehood, the settler colonial health care infrastructure became the foundation of the Israeli health care system. The state supported the sick funds and mandated them to provide health insurance and services to its population, along with medical education and training. These sick funds were motivated by Zionist socialist ideology and oriented toward the Jewish population.^60^ The British-run hospitals became governmental hospitals in the new Israeli state, and thus the young settler colony immediately had a sophisticated health care system, consisting of four nonprofit health care providers, hospitals established by the Zionist movement before 1948, and governmental hospitals. The model drawing from the German Bismarck model of having a small number of health care providers and insurance bodies that are nonprofit and enjoy state support and regulation, with an element of “healthy” competition between the sick funds, allowed for the development of an efficient and cost-effective health service with welfare state elements.^82^

The sick funds thrived as a result of state support and subsidies and had a significant role in providing coverage and insurance to the waves of Jewish immigrants.^60^ They were able to absorb immense challenges as the population of the state of Israel doubled in size within the first few years following the Nakba.^82^ While the Israeli health care system is considered extremely efficient and cost-effective,^83^ it suffers from major racial inequities and inequalities affecting Palestinian citizens of Israel.^84^ These inequities are not a mere coincidence or the result of a lack of equal distribution of resources but rather are deeply embedded in the state's historical and political foundations.^34^

### Health Care of the Palestinian Citizens of Israel

After the Nakba, Palestinians inside the Green Line transformed from a majority to a racialized minority in their homeland. They came to be seen as aliens and even as a “security” risk to the settler state.^78^ After their minoritization^76^ the Palestinian citizens in Israel were subjected to oppressive military rule (from 1948–1966) with severe restrictions on their freedom of movement, residence, denial of the right of displaced Palestinians to return, and discrimination in all aspects of life.^78^ The Ministry of Minority Affairs governed this population, and the medical services in Palestinian towns were coordinated with the military authority.^60^ Palestinian citizens were certainly not in the vision of the Zionist sick funds and had to rely on health care from a government that viewed them with suspicion and as part of an enemy population upon which oppressive military rule was imposed.^77^ Thus, in the first years of the state, Palestinian citizens faced a medical vacuum; with the expulsion of the Palestinian urban middle class, the number of Palestinian physicians dropped from 200 before 1948 to 20 thereafter. The British Mandate services were stopped, and the Jewish sick funds did not cover the Arab Palestinian population.^54,60^ In the early 1950s there were a mere eight doctors working in the office of Israeli Medical Services to the Minorities, rotating between clinics of the Ministry of Health in towns and villages.

By 1956, eight years after the Israeli declaration of statehood, and as the state had more than doubled its population, most Palestinian villages still had no physician attendance at all. Meanwhile, the state was providing health care for the arriving waves of Jewish settlers. Eventually, the numbers of clinics in the Palestinian towns increased and after the end of Israeli military rule, more Palestinians could leave their towns for work and joined the Jewish labor union, becoming eligible for its medical services.^34^ Services to the Palestinians in Israel gradually moved to the Ministry of Health and the sick funds during the 1960s and 1970s. Yet, the establishment of sick fund clinics in Palestinian towns were offered not from a standpoint of a country's duty to its citizens but as a reward or benefit of joining the leading political party Mapai, or as election promises that were sometimes withheld if citizens voted for other parties, such as the case of the communist party in Nazareth.^60^

Palestinians still were clearly not the target audience for the burgeoning Zionist Israeli health care system, and this was demonstrated through explicit policies. In most Jewish towns, for example, land for building clinics was allocated by the Zionist settlement institutions while in Palestinian towns, it had to be donated by the municipalities from the *Waqf* land, belonging to the Muslim or Christian land authority.^60^ As Nira Reiss argued, “The Israeli Ministry of Health activities were constrained by the fact that in Arab communities, health facilities were not planned and funded, as in most Jewish settlements, as a part of a masterplan for settlement under the auspices of the settling agencies”.^60^ As Reiss described, much of the budgets for clinics came from Zionist bodies overseas. Palestinians in Israel were overtly excluded from most of these programs.^60^ This status showcases the stark difference between viewing access to health as a national value and priority, funded and granted by state and non-state actors, versus viewing it as an inevitable burden, outsourced to the community and municipality itself to find and allocate land.

In fact, before Israel passed the 1994 National Health Insurance Law, access to the sick funds was based on membership in the different Zionist labor unions, most importantly the Histadrut. In 1977, while 97 percent of the Israeli Jewish population had health insurance, only 71 percent of Palestinians did.^60^ Only after the 1994 law passed were Palestinian citizens in Israel or residents of Jerusalem eligible for national health insurance and covered by those health care services.^34^ Currently, more than thirty years after having access to national health insurance in what is considered a robust health care system, and despite having great advancement compared to earlier times with a massive increase in the number of Palestinian health care workers^85^ and increased access to health care, health equity is far from being achieved.

Palestinian citizens of Israel continue to have a shorter life expectancy, face higher rates of infant mortality, and suffer from a significantly higher burden of disease compared to Jewish Israelis.^76^ In 2019, the U.N. Committee on the Elimination of Racial Discrimination (CERD) called on Israel to “take concrete measures to improve the health status of the Palestinian and Bedouin populations” inside the Green Line and to “eradicate all forms of segregation between Jewish and non-Jewish communities and any such policies or practices that severely and disproportionately affect the Palestinian population in Israel proper and in the Occupied Palestinian Territory”.^86^ Various modes of structural^87^ and interpersonal racism^88,89^ dominate the Israeli health care system, hampering access to health care facilities for Palestinians^90^ who face explicit racist tropes,^91^ separation in some hospital wards,^92^ denial of insurance to families where one partner is from the occupied Palestinian territory,^93^ and overt signs of militarism.^85^ The Israeli health care system currently employs a large number of Palestinian citizens of Israel, some in high-ranking positions, and this fact is often used as an indicator of successful coexistence. However, the majority of those workers must operate as cautious guests in a system that silences, and sometimes punishes, genuine discussion on issues of apartheid, racism, and Palestinian rights.^85,88^

### Health Care of Palestinians in Occupied East Jerusalem

The Israeli military occupation of east Jerusalem since 1967 operated differently than that of the rest of the West Bank and the Gaza Strip. Israel illegally annexed occupied East Jerusalem as part of materializing the Zionist ethos of Jerusalem as the “eternal and undivided capital of the Jewish people”.^94^ Palestinians in Jerusalem received a special status and identification card as residents, different than that of the West Bank and the Gaza Strip or that of Palestinians citizens in Israel.^69,81^ Israel has invested great efforts in achieving a Jewish demographic majority in Jerusalem, through explicit master plans for the city, and in enforcing a Jewish character on Jerusalem in violation of the city's status under international law.^95^

This all has come concurrently with an attack on Palestinian livelihoods and presence in the city through forcible transfer, including house demolitions,^96^ intense policing, racially-delineated planning, zoning, and educational policies, and the revocation of residency rights for vague grounds of not “maintaining a connection” with the city.^97^ Palestinians in Jerusalem, while forced to pay taxes to the occupying power, receive fewer and less consistent services, essentially subjected to both “organized violence and organized abandonment”.^98,99^ They face violent and racist policing and the occupation's oppressive “security” apparatus,^100^ while being deprived of services such as welfare, education, and employment.^101^ Health care, like other services, is situated within this larger landscape. While Palestinian residents of Jerusalem were included in the Israeli national health insurance, the various sick funds that are in charge of delivering health care in an equitable and timely manner outsource their services to different profit-seeking contractors that employ unqualified and unlicensed staff against regulations, compromising the quality and accessibility of health care.^69^ Thus, while Palestinians in Jerusalem technically have access to the Israeli health care system, their systematic oppression under Israeli occupation and apartheid, being crowded into fragmented and impoverished neighborhoods, some of them separated from the rest of the city by the wall and military checkpoints, some out of reach to Israeli ambulances, hampers access to care in an adequate and timely manner.^69^

While secondary and tertiary care is covered in hospitals, Palestinians report the unnerving experience of receiving care from a system that regards them as the “other” or a threat.^102^ This is most illustrated in the biopolitics of birth as Palestinian women are forced to cross Israeli military checkpoints if they live beyond the wall in order to give birth in a Jerusalem hospital so that their children receive a Jerusalem residency.^103^ Furthermore, health insurance for Jerusalemites remains precarious. Their residency can be revoked if they fail to continuously prove that Jerusalem is their “center of life” or as part of other punitive measures such as if they are deemed to have breached “allegiance” to Israel, even though under international law the occupied population may not be compelled to swear allegiance to the occupying power.^104^ Palestinian Jerusalem residents also cannot extend their national insurance to their spouses or children if they marry Palestinians from the West Bank or Gaza Strip.^97^

While Jerusalem is home to the East Jerusalem Hospital Network that was historically the center of specialized care for Palestinians in the West Bank and Gaza Strip, these hospitals are continuously de-developed, chronically neglected and underfunded,^105^ and sometimes raided by the Israeli occupying forces, with detrimental effects on the lives of patients and health care workers’ ability to deliver care.^106^ Israeli restrictions on freedom of movement between Jerusalem and the rest of the occupied Palestinian territory constitutes another major obstacle to patients and staff in those hospitals, as Palestinians in the West Bank and Gaza Strip must apply for Israeli-issued permits—which are often delayed or denied—to utilize the services of these hospitals.^107^

### Health Care to Israeli Settlers in the Occupied Palestinian Territory

When Israel occupied the West Bank, including east Jerusalem, the Gaza Strip, the Egyptian Sinai Peninsula, and the Syrian Jawlan (Golan) in 1967, Israeli settlers, supported by the state, started establishing illegal settlements and outposts within those occupied territories.^79^ At this point the Israeli health care system was no longer confined to the Green Line, the unofficial frontier distinguishing pre-1967 Israel from the occupied territories. It instead followed those settlers and established clinics^108,109^ and, more recently, a medical school^110^ and plans for a hospital within the settlements.^111^

Because enrollment in those health services required Israeli citizenship and membership in the Jewish labor unions, most Palestinians are inherently excluded from membership and services. The health care system thus transcends the defined state itself and follows settlers as they establish sovereignty, even in occupied territory contrary to international law, delineating the border between settler and native and shaping the contours of medical apartheid. Every Jewish Israeli, whether living in a remote illegal settlement in the hills of the West Bank or in Tel Aviv, has access to the Israeli health care system. Palestinian access, on the other hand, follows hierarchies of identification cards and freedom of movement restrictions,^81,87^ as detailed below.

The number of settlers in the West Bank, including occupied east Jerusalem, has grown significantly over the years to reach more than 700,000.^112^ Health care, similar to other life-sustaining infrastructure, follows those settlers to establish facts on the ground for more than five decades. Jewish-only settler communities enjoy access to clinics and medical facilities inside both the West Bank and the Green Line.^108^ That access to those services is denied to the neighboring Palestinian communities, despite Israel being the only sovereign entity across the entirety of the territory, is perhaps the most blatant sign of medical apartheid. The expanding illegal settlements, with their theft of Palestinian land, water,^113^ and increased settler violence and pogroms against Palestinian communities,^114^ create “chronic war-like conditions” with devastating effects on Palestinian population health^115^ and is an essential tool in the further fragmentation of Palestinian geography.

## The Palestinian Health Care System After 1948

### 1948–1967

In 1949, the U.N. General Assembly established UNRWA to assist the Palestinian refugees who were forcibly expelled through the establishment of the Israeli state to the West Bank, Gaza Strip, Jordan, Lebanon, or Syria.^116^ Unlike short-term humanitarian and relief intervention programs in other war settings, UNRWA was, and continues to be, a major player in health care provision for Palestinian refugees outside and inside Mandate Palestine. It operates wide-ranging, state-like activities, including the provision of primary health care, education, housing, and employment on a large scale.^117^

### 1967–1993

Following the war in 1967, Israel occupied East Jerusalem and the rest of the West Bank, the Gaza Strip, the Egyptian Sinai Peninsula, and the Syrian Jawlan,^118^ thus taking on the obligations of an occupying power in line with the Fourth Geneva Convention of 1949.^119^ The already-weak Palestinian health care system in the occupied territory was now administered by the Israeli Ministry of Defense and, notably, not the Ministry of Health, although there was some supervision from Israeli physicians.^51^ While some policies were standardized between the fragmented Palestinian territories, each had different chief medical officers and followed different protocols of medical practice. Most of the staff on the ground were Palestinian, although all high-level planning was conducted by Israelis. Similar to the Ottoman, British, Egyptian, and Jordanian administrations of the Palestinians, the Israelis focused on infectious disease containment and primary care, with very little investment in secondary and tertiary care.

While the health system in Israel was being heavily developed, the health system of Palestinians in the occupied Palestinian territory was neglected and poorly funded during the initial period of direct Israeli military occupation between 1967 and 1993, with a lack of investment in material and human resources. As Palestinian public health scholar Rita Giacaman and Jad Ishaq wrote in 1977, the annual budget of one Israeli hospital was over three times the budget of all governmental hospitals in the West Bank, and the budget per patient was over ten times higher in Israeli hospitals compared to West Bank hospitals.^120,121^ This de-development^b^ of health services, including the restrictions on provision of medical licenses and the denial of permits for building new hospitals or expanding existing ones,^121^ created a Palestinian dependence on Israeli health care providers for secondary and tertiary care,^123^ which has continued—medical referrals still make up a full three quarters of nonwage health spending.^124^ By 1992, the vast majority of the budget for Palestinian health care went to operating expenses; only 10 percent was devoted to development.

However, Palestinians in this period were not passive subjects; they attempted to create fee-for-service private health facilities, despite the restrictions increasingly imposed by the Israeli occupation. However, serious development of a parallel health system was impossible. In the absence of the development of a formal health system, health professionals established a network of popular health committees^125^ and community-run health care services, such as the Palestine Red Crescent Society (PRCS;^126^), the Palestinian Medical Relief Society (PMRS;^127^), the Health Work Committees (HWC), and private health services that continue to operate to this day.^120^

### 1993–Present

In the early 1990s negotiations between Israel and the Palestine Liberation Organization (PLO) eventually culminated in the adoption of the Oslo Accords, a series of agreements that established the Palestinian Authority (PA), which was meant to serve as the structure of a Palestinian government that Israel would transfer administrative power to Palestinian entities and, after an interim period, lead to the creation of a Palestinian state. The first semblance of Palestinian self-governance was enacted in the Gaza–Jericho Agreement signed in 1994, which gave the newly formed PA command of internal affairs, such as health and education.^128^

The newly established Palestinian Ministry of Health inherited a patchwork of health services composed of under-resourced, weak, and fragmented health facilities and attempts of Palestinian health professionals to set up independent health care facilities^129^ after a century of power transfers and lack of investment. Further, the broader mechanisms of control, including of Palestinian borders and the movement of people and goods, remained under Israel's effective control, rendering them a significant actor in all aspects of Palestinian health.

The post-Oslo years did not bring an end to the occupation, let alone an independent sovereign Palestinian state.^130^ On the contrary, Israel continued entrenching its occupation and apartheid regime,^131^ with the expansion of illegal settlements and an expanding system of physical and bureaucratic movement restrictions, including checkpoints and the Separation Wall in the West Bank, the illegal siege and blockade of the Gaza Strip since 2007, and a complex system of Israeli-issued permits for movement of Palestinians across the West Bank and between Gaza and the West Bank. These permits included those needed for entry into occupied East Jerusalem, where the most advanced specialized Palestinian hospitals are located.^79^

While the Oslo Accords did not culminate in a Palestinian state, their “interim” status became the permanent reality for Palestinians in the West Bank. The Accords subdivided the West Bank into areas A, B, and C with purported full Palestinian civil and security control over Area A, Palestinian civil and Israeli military control over Area B, and full Israeli control over Area C, which makes up the majority of the West Bank (60%). This division further weakens the ability of the PA to reach and expand health services to these areas as construction and infrastructural work in Area C requires an Israeli-issued permit, the majority of which are denied to Palestinians.^132^

Furthermore, the construction of illegal Israeli settlements included segregated road systems, infrastructure, and accompanying settler violence and pogroms against Palestinians, such as attacks on ambulances,^133^ constituting a risk to Palestinian health care staff and patients in these areas. Palestinians across the occupied territory are denied their right to sovereignty over their land, water, airspace, ability to import or export, and the ability to interact with the outside world, all of which are tightly controlled by the occupying power. As a result of the Paris Protocol of the Oslo Accords, the Palestinian economy also became subordinated to the Israeli economy. While Israel continues to retain effective control as the occupying power across the occupied Palestinian territory, it has illegally transferred its obligation to the population it occupies by claiming it had ceded its obligations to the non-sovereign PA.^79^ Unlike other countries in the Global South that achieved independence and had the opportunity to establish their own health care systems while still struggling with issues of impoverishment, corruption, and brain drain, the Palestinians, on top of dealing with those challenges, continue to live under a regime of apartheid, occupation, and settler colonialism that governs every aspect of their lives and violates their right to the enjoyment of the highest attainable standard of health.

This reality has resulted in the creation of various health services that are fragmented, weak, and highly-dependent on Israel, both for services provided in their own territory^134^ and those for which they are allowed to travel to receive.^135^ Today, Palestinians in the occupied Palestinian territory receive health care from a patchwork of providers, including UNRWA, the Palestinian Ministry of Health, non-governmental organizations (NGOs), and a growing private sector.^136^

Furthermore, the unmaking of Palestinian statehood and sovereignty has created a reality of a pseudo-state without a social contract between citizens and government, meaning there is no active and free democratic debate about what the obligations of the State of Palestine are toward its tax-paying citizens and what services are expected in return for taxes or international aid. This is most blatant when it comes to security, as the PA cannot provide Palestinians any security against the attacks of the Israeli army or settlers, but also in terms of the provision of health care, education, adequate housing, water, and electricity, among others. Palestinians are left to maneuver their way through the health services they can reach from diverse providers based on location, class, and connections.

The current landscape of Palestinian health services is characterized by:

1. A weak primary health care system: Some primary health care clinics are operated by the Palestinian Ministry of Health and available to Palestinians with governmental health insurance (those working in government or security positions, families of political prisoners, families of martyrs, and those who join this health insurance voluntarily). But they are under-resourced and understaffed, and plagued with inefficiency, cronyism, and corruption.^136^ UNRWA clinics also provide primary health care services in the West Bank, Gaza Strip, and in neighboring countries where Palestinian refugee camps are housed. The primary health services that are the cornerstone of modern public health in disease prevention and screening are thus not well-defined, weak, and inefficient. There is no standardized and trained role of the physician, nurse, nutritionist, or other health workers in family medicine, pediatrics, or primary services in general. An exception here are the childhood vaccine programs, which have extensive coverage.
2. Scattered secondary health care providers: Palestinians also seek health care providers when they face a medical condition that requires treatment. The private sector dominates the landscape with either individual physicians or small-scale clinics offering specific services or consultation, treatment, and follow up.^137^ The Ministry of Health, NGOs, and the medical military services also provide secondary health care services and hospital services to varying degrees.^138^
3. Limited tertiary services: The marginalization of Jerusalem as the capital city and the center of tertiary hospitals and specialized services and the increasing restrictions on the movement of patients, staff,^139^ and medical equipment to Jerusalem due to the wall, siege, checkpoints, and permit regime has significantly hampered access to Jerusalem and has led to the duplication and fragmentation of services in a relatively small geographical area. Patients from within the occupied territories are often referred to Israel, Jordan, or other countries for complex tertiary care—often legitimately and sometimes based on cronyism, which exhausts nearly 40 percent of the Palestinian Authority's Ministry of Health budget, and is a major reason for the chronic starvation for needed funds.^140^
4. Restriction of movement: The major restrictions on movement and the poor implementation and regulation of medical referrals abroad combined with corruption and cronyism^136^ has hindered the ability to plan and expand a network of primary, secondary, and tertiary services,^140^ with substantial financial benefit incurred by the Israeli medical system.

This highly fragmented, aid-dependent system is strangled by the Israeli military occupation in multiple ways^141^ and is often stranded in relief and emergency mode, unable to conduct long-term planning.^136^ Decades of preexisting health disparities against Palestinians under Israel's effective control became particularly visible in the context of the COVID-19 pandemic, where Israel disavowed its legal obligations by refusing to vaccinate Palestinians under occupation while delivering vaccines to illegal settlers in the same territory.^119^ Israeli authorities also undermined Palestinian responses to the pandemic^142^ by shutting down a testing center in occupied east Jerusalem,^101,105^ delays in testing for COVID-19 inside the Green Line,^143^ bombing testing facilities in the Gaza Strip,^144^ and even delaying a shipment of vaccines from the West Bank to Gaza.^145^ The obvious discrepancy in Israel's response to the pandemic blatantly made clear the two-tiered system of health available for Palestinians under Israel's effective control, offering a distinct view of what medical apartheid looks like in practice.^146,147^

Despite increased investment over time, the Palestinian healthcare system has only been able to improve partially and continues to stagnate in many areas. The systemic weakening of the Palestinian health system and the restriction on equipment and raw materials via the “dual use”^c^ condition^148,149^ set in the 1994 Paris Protocol make it necessary to outsource services outside the system to Israel or the private sector, maintaining and reinforcing a state of colonial dependency. Aside from Israeli control over the permits,^150^ which arbitrarily restrict Palestinians’ access to care, the PA is tasked with paying for the patients Israel approves for transfer to Israeli facilities. The de-development of the Palestinian economy^122^ affects the ability to invest in human resources as well as structures and infrastructures. Furthermore, the significant portion of the Ministry of Health budget used to pay for referrals could potentially be used to develop the health system further and thereby constitutes a missed opportunity for development.

On top of Israel's prolonged unlawful military occupation^7^ and its apartheid regime, several local factors that are outside the scope of this article, such as corruption, cronyism, prioritizing the preferences of donors over a national agenda, lack of transparency in appointments and accountability within the health care system,^136^ lack of an active role of professional associations in shaping the health care system, the lack of a free and public debate in civil society and newspapers critiquing the PA and its handling of the health services, the Fatah-Hamas rift and its effect on the Ministries of Health in the West Bank and Gaza Strip, and the brain drain continue to weaken the potential of Palestinian health care.

### Siege, Wars, and Genocide in Gaza

Following the 2006 Palestinian elections, when Hamas was elected to power, Israel categorized Gaza as a “hostile” territory and instituted an illegal land, air, and sea blockade, amounting to unlawful collective punishment^104^ that remains to this day.^151^ Shortly thereafter, Hamas and the PA fractured politically, also dividing the government institutions between the West Bank and Gaza Strip.^152^ Along with the delineation of the West Bank into Areas A, B, and C, this separation added to the post-Oslo fragmentation that still continues to accelerate.^118^

The Israeli siege over Gaza did not only control the land, maritime, and airspace of the enclave and the movement of people and goods in an act of collective punishment,^151^ it further impoverished and de-developed the Gaza Strip.^153^ Israel has repeatedly attacked and targeted the Palestinian health care system along with other civilian infrastructures. The attacks on health care include the killing of health care workers and bombing health care facilities and ambulances, including bombing the COVID-19 laboratory during the pandemic.^144,154^ Israel attacks health care across the occupied Palestinian territory, including east Jerusalem^106^ and the West Bank,^133^ yet the scale and magnitude of the attacks in the Gaza Strip after the withdrawal of illegal settlements in 2005 and since implementing the siege in 2007 has been unprecedented. Farhat and colleagues have diagnosed the situation in the Gaza Strip as a “biosphere of war”^155^ with repetitive cycles of military attacks causing a devastating death toll and destruction to health and other infrastructures, only to be followed by a slow phase of reconstruction until a new military assault is launched.^156^ Nicola Perugini and Neve Gordon have coined the term “medical lawfare” to describe the systematic nature of the Israeli attacks on health care facilities and the weaponizing of law to legitimize the destruction of the Palestinian health system, described as “hospital shields” similar to human shields, while blaming the Palestinians for the destruction and thus continuing the settler colonial logic of elimination.^154^

While this article was being written and reviewed, Israel launched its deadliest military campaign on the Gaza Strip since the beginning of its illegal blockade in 2007 and one of the deadliest wars in modern history in what leading human rights organizations and experts have determined is an unfolding genocide.^12,157–159^ On October 7th, 2023, Hamas and other Palestinian armed groups launched an attack on Israeli military bases, towns, and a rave near the Gaza fence. As a result, 1,269 Israelis were killed, including 816 civilians (Several reports have indicated that a mixture of Palestinian and Israeli fire contributed to the death toll).^160,161^ Palestinian militants also abducted over two hundred Israelis and demanded the release of more than 5,000 Palestinian prisoners from Israeli prisons and detention centers.^162^ Israel responded by launching its genocidal war that included unprecedented, indiscriminate bombardment of the besieged enclave, and a further tightening of the siege blocking the entry of food, water, fuel, and medications. Israel's genocide against 2.3 million Palestinians has included the systematic destruction of hospitals and clinics in the Gaza Strip,^44,163^ the killing and injury of hundreds of health care workers, and the kidnapping, arbitrary detention, torture, sexual violence, and enforced disappearance of many other people.^164,165^ In acts of reproductive genocide,^166^ Israel bombed in vitro fertilization clinics and an embryo bank with over 4,000 embryos; neonates were left to decompose as hospitals were attacked and forced to evacuate.^167^ While the current genocide is beyond the scope of this article and cannot be fully addressed by it, it should not be seen as an isolated event but rather a continuation and entrenchment of the Israeli apartheid, settler colonial, and necropolitical policies targeting the Indigenous Palestinian people for elimination.^168–170^ The destruction of the Palestinian health care system, together with all other life-sustaining infrastructure and social determinants of health (water, food production, fuel, electricity, adequate housing, education) and the declared intent to “flatten Gaza” and send it back to the “stone age,” as expressed by Israeli officials,^171,172^ is a clear escalation of the Israeli strategy of elimination by targeting the health system and main determinants of health^173^ and with it the ability of Palestinians to care for each other and sustain life now and in the future. As of the time of writing, Israel has killed more than 64,656 Palestinians^174^ in the Gaza Strip, including more than 20,000 children.^175^ Almost 4,000 children had at least one limb amputated, making the Gaza strip the place with the highest number of child amputees in the world.^176^ The first year of the genocide has caused a 34.9 years’ drop in the life expectancy in the Gaza Strip, almost half of the pre-war level of 75.5.^177^ Between siege, displacement, starvation,^178^ decimation of health care, massive bombardment, debris, and military waste, Israel has turned Gaza into an extermination camp and a graveyard for slow agonizing death, disability, and grief.^179,180^ The genocide is a global health crisis.^168–170^

## Limitations

This article deals primarily with health systems themselves as they developed over time and largely does not consider the settler colonial determinants of health, as they are discussed in-depth elsewhere.^25,87^ In this article we did not detail how specific Palestinian communities are more affected by medical apartheid than others, such as Bedouin communities in Area C, Bedouins in unrecognized villages in the Naqab, and other marginalized and impoverished groups.

## Conclusion

For Palestinians, health equity cannot be achieved simply with aid, development, or even solely the end of Israel's occupation, blockade, or armed conflict. A deep historical analysis suggests that the gaps in Palestinian health care have been created intentionally and systematically over more than a century of Zionist settler colonialism to maintain racial oppression and domination over all “non-Jewish” others^76^ under Israeli control. The adoption of the apartheid framework of analysis and the recognition of Israel as an apartheid state by human rights organizations, scholars, experts, and states around the world is a useful step in naming the regime of oppression targeting Palestinians. Illustrating how apartheid manifests in various sectors of society is necessary to understand why certain outcomes are reported, certain interventions cannot be implemented, and aid does little to build long-term capacity. Apartheid systems do not create inequities by accident—these inequities are created by design. There is no supremacy without inferiority; indeed, equity is a threat to domination. Understanding how systems of medical apartheid develop is not an esoteric exercise but should be seen as one way to understand how fundamental inequities develop in society, who these systems benefit, and what needs to be done to dismantle them.

We argue that it is not enough simply to describe health outcomes as a form of apartheid but that it is necessary to trace the origins of racial oppression and domination among and between communities. Further, the intrinsic connection between health and all other structures must be explored. There is no sector unaffected by population health, and population health also ripples into countless other societal measures, from educational outcomes to economic measures.

Adopting a historical perspective to track the building blocks of medical apartheid reveals what Gilmore has called “landscapes of accumulation and dis-accumulation”.^181^ For Israelis, health and health care accumulate over time with growth of the health care system, improvement, and an expanding expertise and reputation. The Palestinians, on the other hand, are rendered in a constant landscape of dis-accumulation in a system struggling to survive, strangled, attacked, and decimated. Accumulation by dispossession in the apartheid settler colonial state^182^ does not only confer land, water, and capital, but also health.

Health care has always been a tool for members of society to care for each other and to grow, heal, and flourish collectively. To varying degrees depending on the status conferred upon them by Israel, fragmented Palestinians consistently suffer from poorer health outcomes as a result of medical apartheid. They also face systematic attacks on health care, particularly in the context of the ongoing genocide in Gaza, and a denial of their rights to development, return, and self-determination. The apartheid framework allows for consideration of the role that fragmentation plays in oppressing Palestinians as a people, denying them both collective and individual rights, including the full enjoyment of the right to health and its underlying determinants. Health equity and justice can only exist when apartheid is fully dismantled,^32^ Palestinian rights to return and self-determination are realized, and all people live equally and freely between the River and the Sea.

## Notes

a. The trope of transfer of Palestinians to neighboring countries and denying them the right to live on their ancestral homeland is still echoed in the statements of American and Israeli officials during the genocide in Gaza.57

b. We use the term “de-development” as articulated by Sara Roy122 as a structural relationship where a dominant economy not only distorts the development of the subordinate economy but undermines it entirely.

c. The designation of items as “dual-use” means that Israel considers those items to be inherently for civil use but that they can be used for military purposes and thus are either banned or require special permits. This policy has strangled the Palestinian economy, especially in industry, agriculture, and health, and is one of the manifestations of the Israeli control over Palestinian daily life.
